# The Impact of Broadcasters on Consumer’s Intention to Follow Livestream Brand Community

**DOI:** 10.3389/fpsyg.2021.810883

**Published:** 2022-02-03

**Authors:** Wei Wang, Minxue Huang, Shiyong Zheng, Liangtong Lin, Lei Wang

**Affiliations:** ^1^School of Economics and Management, Wuhan University, Wuhan, China; ^2^School of Business, Guilin University of Electronic Technology, Guilin, China; ^3^Management School of Hainan University, Haikou, China

**Keywords:** livestream e-commerce, livestream brand community, motive theory, broadcaster type, intention to follow livestream brand community

## Abstract

As the essence of livestream e-commerce is social commerce, building a livestream brand community and attracting brand followers are the key aspects to achieving sustained revenue. For many companies, inviting celebrities has become a shortcut to attract new followers. Considering the unsustainability and high cost of the celebrity host mode, some companies switched to using their own branded broadcasters to attract followers. However, as branded broadcasters lack a fan base, choosing the suitable broadcaster type has become a challenge in livestream e-commerce. The motivation of consumers to follow brand livestream accounts is mainly to obtain potential value by embedding them in social networks. Therefore, based on motive theory, this research explores how different broadcaster types affect consumer’s intention to follow a livestream brand community. Results from the analysis of secondary data from livestream platforms and two laboratory experiments reveal that (1) celebrities contribute more to consumer’s intention to follow than branded broadcasters, and utilitarian (vs. hedonic) products can strengthen the effect of branded (vs. celebrity) broadcasters on attracting potential followers. (2) Moreover, branded (vs. celebrity) broadcasters can promote consumer’s intention to follow a livestream brand community by satisfying consumer’s need for informational (vs. emotional) value during utilitarian (vs. hedonic) product evaluation. This research analyzes the differential effects of different types of broadcasters on livestream brand community building. The findings can deepen the understanding of the consumer’s behavior of following brand livestream communities and provide companies with suggestions on broadcaster selection in livestream e-commerce.

## Introduction

The spread of COVID-19 is like a catalyst stimulating the development of livestream e-commerce. By December 2020, the size of livestream e-commerce users had reached 388 million, and the market is expected to exceed 2 trillion yuan in 2021 (CNNIC, 2021).^1^ In view of its huge potential profits, companies have started to build livestream platforms or cooperate with third-party platforms to explore the livestream e-commerce market. Unlike traditional e-commerce, the essence of livestream shopping is social commerce. Consumers with common brand interests in livestream shopping gather together in the virtual live room for product information sharing and emotional interaction to satisfy their purchasing needs and emotional needs (Wongkitrungrueng et al., 2020). To obtain sustainable revenue from a livestream community, companies must develop and accumulate their own fans (Tang and Chen, 2020). However, previous studies on livestream shopping mainly focused on the short-term transaction (e.g., sales, purchase intention) (Zhao et al., 2018; Hou et al., 2019; Ma and Mei, 2019) and paid little attention to the long-term value of follower attraction.

This research focuses on new follower attraction, which is the premise of the development of a livestream brand community. Attracting new and retaining existing followers are the two key aspects that can guarantee the long-term survival of a livestream community, because the continuous participation of followers can maintain group vitality, promote consumers’ brand evaluation and purchase intention, and expand the influence of a brand (Beukeboom et al., 2015; Casaló et al., 2017; Tang and Chen, 2020). As celebrities possess their own fan base, choosing a celebrity as a broadcaster may be a shortcut to increase consumers’ intention to follow a livestream brand account (Jin and Phua, 2014; Hou et al., 2019). However, some companies are concerned about the high cost of celebrities and find transforming the fans of a celebrity into the fans of a brand challenging. As a result, some companies began adopting their own branded broadcasters to sell their products. Although branded broadcasters may be helpful in brand fan cultivation and accumulation, their publicity is limited. Therefore, enterprises are facing a dilemma in the selection of broadcasters.

To solve the dilemma, we must discuss why consumers follow livestream brand communities. Consumers’ following behavior has two main motivations, that is, informational value appeal and emotional value appeal (Pai and Arnott, 2013; Lim and Kumar, 2019). When the two needs are satisfied, consumers may follow an online brand community and build a relationship with the brand to obtain increased value in the long run (Casaló et al., 2017; Tang and Chen, 2020; Bharadwaj et al., 2021). As a guide who assisting consumers’ interaction and consumption, broadcasters can generate value for an online community (Casaló et al., 2017). We assume that different types of broadcasters have diverse value and thus can satisfy consumers’ various needs, which can help to attract consumers to become fans of the livestream brand community. Celebrity broadcasters are generally attractive and can excite and make consumers happy through positive affective experiences (Pai and Arnott, 2013; Hou et al., 2019). Thus, celebrity broadcasters can influence newcomers with emotional needs to follow livestreams. Branded broadcasters can generally provide consumers with professional and reliable product information. Therefore, branded broadcasters can attract consumers who are concerned about informational value to follow a community. Consumers have various needs for different products. For instance, they are highly concerned about the emotional need for hedonic products (Asano et al., 2020). Thus, celebrity broadcasters may be the best choice, as they can touch on entertainment topics. By contrast, consumers who are highly concerned about product information and quality may prefer branded broadcasters for professional guidance.

In summary, this paper will try to solve the following questions: (1) Which type of broadcaster (celebrity vs. branded broadcaster) can attract potential followers better? (2) What is the mechanism behind the main effect? (3) Is there a boundary (e.g., product type) influencing the effect of broadcaster type? To study these questions, we have conducted three studies in this paper. Study 1 provides an initial examination of how broadcaster type and product type influence the growth of live brand community size by analyzing 461 livestream sessions from a livestream platform. Study 2 verifies the main effect of the broadcaster type and the moderation effect of product type on consumer’s intention to follow a livestream brand community through a laboratory experiment controlling underlying confounding factors. Finally, Study 3 retests the main and moderating effects and verifies the mechanism of motivations using a laboratory experiment.

This research may provide the following contributions. First, since we have compared and analyzed the different impacts of celebrity and branded broadcasters on the construction of brand livestream communities, the paper may broaden the understanding of how the types of broadcasters might lead to different outputs. Second, the paper would deepen the understanding of consumers’ livestream brand community following behavior since it has provided insights into the different needs of consumers in online community participation based on motive theories. Third, it may enrich the boundary of the main effect of broadcaster types. Firms should select appropriate broadcasters to match consumers’ value needs for different product types to improve the broadcaster’s effects.

## Related Literature and Hypotheses

### Livestream Brand Community

Existing research mainly considers livestream e-commerce as a model that combines livestream with e-commerce, where companies (sellers) engage in one-to-many real-time social interactions with consumers through a live webcasting platform to realize product sales (Wongkitrungrueng et al., 2020). Compared to traditional e-commerce, it has several social advantages (Akram et al., 2020). First, broadcasters in live e-commerce are not virtual as in traditional e-commerce that they expose their faces and personalities to the consumer community to enhance authenticity and promote trust and intimacy between them and consumers (Akram et al., 2017; Hilvert-Bruce et al., 2018; Wongkitrungrueng and Assarut, 2020; Lu and Chen, 2021). Second, the real-time interaction between broadcasters and consumers could reinforce the interactive experience of consumers (Wongkitrungrueng and Assarut, 2020; Xu et al., 2020; Zhang et al., 2020; Fei et al., 2021). Third, the display of comments and number of viewers could increase the perceived social presence of consumers (Wongkitrungrueng and Assarut, 2020; Ming et al., 2021).

However, the social essence of live e-commerce is not only reflected in the aspects above but also in its community nature. Fans gather together in live rooms, engage in informational and emotional interaction, and make purchases under the guidance of broadcasters. They gradually achieve a community consensus and a community identity (Schau et al., 2009; Jeong et al., 2021). From the perspective of the community, some scholars have explored the influence of consumers’ perceived value on their engagement and purchase intention based on use and gratifications approach and motive theory (Hilvert-Bruce et al., 2018; Zhang et al., 2019; Park and Lin, 2020; Wongkitrungrueng and Assarut, 2020). Furthermore, some scholars have explored the impact of perceived usability of the livestream technology on consumers’ purchase intentions based on the technology acceptance model (Sun et al., 2019). Based on social facilitation theory and psychological arousal theory, some scholars have also studied how perceived social presence in virtual scenarios might promote consumers’ purchase intentions in live shopping (Ou et al., 2014; Martini, 2018; Wang et al., 2021). Other relevant literature is summarized in Table 1.

**TABLE 1:** Main literature about livestreaming shopping in recent 3 years.

Articles	Independent variable	Dependent variable	Theory	Data	Broadcaster type	
					Branded broadcaster	celebrity Broadcaster
Hilvert-Bruce et al. (2018)	Motives	Participation intention	Use and Gratifications theory	Survey	—	—
Sun et al. (2019)	Ease of use	Purchase intention	Technology acceptance model	Survey	—	—
Hou et al. (2019)	Traits	Watching intention Purchase intention	Use and Gratifications theory	Secondary data	√	√
Wongkitrungrueng and Assarut (2020)	Perceived value	Participation intention	Motive theory	Survey	√	
Park and Lin (2020)	Celebrity and contents	Purchase intention	Source credibility theory	Survey		√
Zhang et al. (2020)	Contents	Purchase intention	Explanatory level theory	Secondary data	√	
Xu et al. (2020)	Contextual and Environmental stimuli effects	Hedonic consumption, impulsive consumption, and social sharing	SOR framework	Survey	—	—
Fei et al. (2021)	Social cues (Herding message and Interaction text)	Purchase intention	SOR theory	Laboratory experiment	—	—
Meng et al. (2021)	Celebrity	Purchase intention	Emotion contagion theory	Survey and Secondary data	—	√
Lu and Chen (2021)	Broadcasters’ physical characteristics and Instant interaction	Purchase intention	Signaling theory	Survey	—	—
Bharadwaj et al. (2021)	Emotional display	Sales	Emotion contagion theory	Secondary data	—	—
Lin et al. (2021)	Broadcaster emotion	Tipping	Emotion contagion theory	Secondary data	—	√
Li et al. (2021)	Technical and Social factors	User stickiness for platform	Attachment theory	Survey	—	—
Ming et al. (2021)	Social presence	Impulse purchase	SOR theory	Survey	—	—
***This study***	*Broadcaster type*	*Intention to follow livestream brand community and cumulant of new followers*	*Motive theory*	*Secondary data and Laboratory experiments*	√	√

In summary, previous studies have mainly focused on short-term individual interactions and purchase intentions (see Table 1), but the discussion on the long-term sustained development of livestream communities is limited. The mass of followers is one of the most important indicators for community development evaluation (Tsai et al., 2012; Casaló et al., 2017). Therefore, in this study, we discuss the factors influencing follower attraction.

### The Influence of Broadcaster on Consumers’ Intention to Follow Livestream Brand Community

Intention to follow a livestream brand community refers to a consumer’s tendency to hope to receive livestream messages and updates automatically (Casaló et al., 2017; Tang and Chen, 2020). Although many other important interactive behaviors (e.g., likes and comments) can reflect consumers’ satisfaction of a livestream brand community, following behavior indicates their decision to establish a deep, interactive, and trading relationship with the brand (Mahmoud et al., 2021). From the perspective of firms, sustained financial benefits and brand influence can be realized only if they can attract and maintain a sufficient number of followers. Consumers’ commitment increases after following a livestream brand community (Zhang et al., 2018), and followers are likely to create positive word of mouth (WOM; Kim et al., 2014). Owing to the premise and important role of follower attraction, many companies offer economic rewards to increase their followers (Pöyry et al., 2013). In livestream e-commerce, companies generally take advantage of broadcasters to attract new followers.

In livestream e-commerce, two common types of broadcasters are celebrity and branded broadcasters. Celebrity broadcasters are individuals who are widely known by the public for their outstanding achievements in domains unrelated to a brand or product (Sertoglu et al., 2014; Escalas and Bettman, 2017). Celebrities generally broadcast on their personal livestream channel or are invited by firms as broadcasters to attract consumers. Meanwhile, branded broadcasters are non-celebrities and, similar to salespersons, hired by firms to sell products in a livestream brand community (Lou et al., 2019). Branded broadcasters are generally not as famous as celebrities and work for only one brand channel to increase brand followers and sales.

Previous studies focused mainly on the influence of celebrity broadcasters’ personal traits on consumers’ purchase intentions (Hou et al., 2019; Ma and Mei, 2019) and paid little attention to the follower attraction effectiveness of different broadcaster types. Consumers may evaluate a brand *via* broadcasters’ perceptions (source of information) when deciding to follow a livestream brand account (meaning transfer process) (De Veirman et al., 2019). Based on the source credibility model and source attractiveness model, consumers may consider three dimensions for evaluating broadcasters, namely, expertise, trustworthiness, and attractiveness (Erdogan, 1999).

Celebrity and branded broadcasters may have different strengths in terms of attractiveness and expertise, which may have different effects on consumers’ intention to follow a livestream brand community. Branded broadcasters generally have more expertise in products than celebrities. In addition, branded broadcasters undergo professional training and are equipped with solid expertise in production processes, materials, and usages and can provide consumers with reliable, professional, and comprehensive product information (Tuncdogan et al., 2017). However, compared with celebrity broadcasters, branded broadcasters may have less competitiveness in terms of attractiveness (Saeed et al., 2014). Thus, consumers may obtain limited hedonic and experiential value from the interaction with branded broadcasters (Lin et al., 2021). Meanwhile, celebrity broadcasters generally have an attractive appearance and character, which may bring joyful and exciting experiences to consumers (Hou et al., 2019; Zhu et al., 2021). Despite having less product knowledge and brand awareness than branded broadcasters, celebrity broadcasters also undergo preparations to be able to answer basic questions from consumers and arrange promotion activities (Wongkitrungrueng and Assarut, 2020).

Previous studies showed that significant differences in trustworthiness may not exist between the two types of anchors in livestream settings. On the one hand, celebrity endorsements can improve consumers’ perceived trustworthiness (Erdogan, 1999), but the high sponsorship cost of celebrity attendance may offset their persuasive effect (Kim et al., 2021). On the other hand, branded broadcasters can increase consumers’ perceived trustworthiness through their professional product knowledge and expertise in problem solving; however, their employee role representing corporate interests may also reduce consumers’ perceived trustworthiness (Lou et al., 2019; Kim et al., 2021). Based on the discussion on the three dimensions, it can be seen that celebrity broadcasters cannot only meet consumers’ basic product information needs but also strengthen consumers’ livestream shopping experience by providing them with hedonic experience. These positive cognitions and affects toward broadcasters may further transfer to products and brands, which is referred to as *meaning transfer* (De Veirman et al., 2019). Therefore, they can effectively promote consumers’ willingness to maintain a long-term relationship with the livestream brand community. Thus, the following hypothesis is proposed:

**H1**: In livestream shopping, the broadcaster will have an impact on consumers’ intention to follow a livestream brand community. Additionally, celebrity broadcasters are more likely to promote consumers’ intention to follow a livestream brand community than branded broadcasters.

### Moderating Effect of Product Type

Due to the social and transactional nature of livestream shopping, the factors affecting consumers’ intention to follow a livestream brand community include not only the value brought by broadcasters, but also the product itself. Utilitarian products mainly refer to the products or services that can meet consumers’ instrumental and functional needs (Asano et al., 2020; Garrido-Morgado et al., 2021). Consumers tend to pay more attention to the functional value of utilitarian products and are more likely to care about the professionalism, comprehensiveness, and reliability of the product information. Branded broadcasters generally have a more comprehensive and in-depth understanding of the product information. Therefore, branded broadcasters can provide high-quality information to consumers with different knowledge levels and promote their cognition of the broadcaster’s expertise to transfer to a product and brand, which can ultimately lead to high consumer intention to follow the livestream brand community. Although celebrities also need to be familiar with basic product knowledge and skills, their knowledge depth and breadth are relatively limited for utilitarian products. In other words, they are less likely to lead in-depth product discussions. Additionally, it is also difficult for them to meet consumers’ expectations for expertise in product introduction. Therefore, consumers may prefer branded broadcasters over celebrities for utilitarian products and follow a livestream brand community when the broadcaster is from the firm.

By contrast, consumers tend to focus more on the affective and experiential value in hedonic product purchases (Chitturi et al., 2008; Asano et al., 2020). As hedonic products are difficult to be evaluated by parameters or specific standards, consumers tend to rely more on broadcasters for the evaluation and decision-making. In addition, celebrity broadcasters may enhance the consumers’ interactions and purchase experiences of hedonic goods. Celebrity broadcasters generally have stronger attractiveness that they can enliven the atmosphere of livestream rooms and strengthen the interactive experience of consumers. Consumers who are willing to acquire more experiential and affective value will then become the brand followers. Furthermore, celebrities generally have a high social status that consumers can join the brand community and become brand fans to keep in line with their favorite celebrities so as to realize their demand for self-enhancement (Raghunathan and Corfman, 2006; Meng et al., 2021). Although branded broadcasters will also introduce the experiential aspect of hedonic products, they have certain limitations compared with celebrities. For example, they are generally not as attractive as celebrities and lack of halo effects. Therefore, consumers can obtain more emotional satisfaction from celebrity broadcasters regarding hedonic products. Thus, they are more willing to establish long-term relationships with the brand and become brand fans. Therefore, the following hypotheses are proposed:

**H2:** Product type moderates the effect of broadcaster type on consumers’ intention to follow a livestream brand community.

**H2a:** Consumers are more likely to follow the livestream brand community when branded broadcasters recommend utilitarian products.

**H2b:** Consumers are more likely to follow the livestream brand community when celebrity broadcasters recommend hedonic products.

### Mediating Effect of Motivation

Uses and gratifications theory states why people would access a medium to satisfy some of their specific motivations (Dolan et al., 2015). Individual motivations mainly include external motivation and internal motivation. In terms of external motivation, some scholars have identified the positive impact of economic incentives, such as coupons and lotteries, on consumers’ engagement with the virtual brand community (Lim and Kumar, 2019). External motivations such as economic incentives play a very limited role in the long run, weakening internal motivations (Plant and Devine, 1998). Therefore, many scholars pay more attention to the satisfaction of internal motivations. Scholars have identified three internal motivations: the functional value, hedonic value, and social value (Lim and Kumar, 2019). In live e-commerce, the functional value mainly refers to the informational aspect of the product, technologies, producing process, usage, brand culture, and so on (Lim and Kumar, 2019). These contents can satisfy consumers’ instrumental demands by enriching their product knowledge and enhancing their awareness and brand trust. The hedonic value mainly refers to the good feelings such as happiness, pleasance, and interesting experience that consumers have in the participation of brand communities (Pai and Arnott, 2013). These positive emotions will motivate consumers to continue participating in the brand community. Finally, the social value mainly refers to the achievement, reputation, status, and self-efficacy that consumers gain from long-term community participation and also the interaction with other members (Pai and Arnott, 2013; Lim and Kumar, 2019). Social values are generally discussed in communities of relationship (e.g., Weibo, Twitter) and interest (e.g., Zhihu, Douban, Tiktok) (Kim and Song, 2016; Yilmaz, 2016). But this study mainly discusses the consumer’s behaviors on a transactional livestream platform (Taobao livestream platform). So, this study will just focus on the informational and hedonic value to explain the mechanism behind it (Kozlenkova et al., 2017; Li et al., 2021).

In live shopping, branded broadcasters tend to provide reliable and high-quality product information for consumers (Lou et al., 2019; Wongkitrungrueng and Assarut, 2020). Thus, they can promote newcomers, especially those who concern with product information to follow the brand to gain more functional value in the long run. For utilitarian products, consumers mainly focus on the reliability and quality of product information, so the informative resources provided by branded broadcasters may attract the consumers concerned about the informational value to follow brand livestream shopping (Akram et al., 2021). On the contrary, celebrity broadcasters tend to create more emotional value than branded broadcasters. First, celebrity broadcasters generally have higher personal charisma and attractiveness (Hou et al., 2019). Second, celebrities generally have higher popularity and social status, so consumers can shorten the psychological distance with them and meet the need for self-identification construction by joining the community (Ashforth et al., 2008). Third, as public figures, most celebrities have strong social skills to establish a good relationship with consumers. Fourth, celebrities are usually the focus of public attention, which helps to attract consumers’ interest and participation and eventually enhances emotional experiences of consumers. Therefore, they are more likely to promote newcomers who concern with emotional value to follow the brand to gain more hedonic value in the long run. Additionally, the effect may be strengthened when making a hedonic product purchase because celebrity broadcaster would match consumers’ emotional value appeals. Thus, we propose the following hypotheses:

**H3**: Both (3a) informational value appeal and (3b) emotional value appeal mediate the effect of broadcaster type on consumers’ intention to follow a livestream brand community, and the mediated relationship is moderated by product type.

**H3a**: Branded broadcasters are more likely to satisfy consumers’ informational value appeal, which will promote consumers’ intention to follow a livestream brand community especially regarding utilitarian products.

**H3b**: Celebrity broadcasters are more likely to satisfy consumers’ emotional value appeal, which will promote consumers’ intention to follow a livestream brand community especially regarding hedonic products.

**FIGURE 1:**
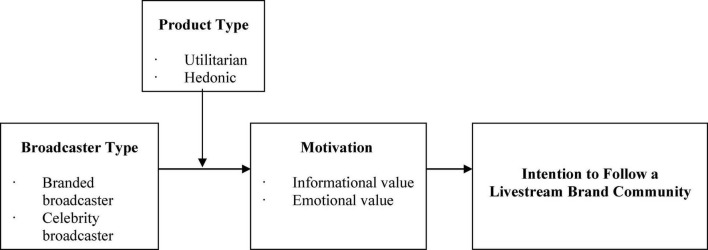
Research framework.

The research framework of this research is shown in Figure 1.

## Study 1: Main Effect and Moderating Effect Test Based on Secondary Data

### Data

To explore the influence of broadcaster types on the new follower attraction, we use the data crawling software Bazhuayu to collect the real livestream data from Taobao, a livestream shopping platform. Taobao is the most influential livestream shopping platform in China, where over 12 thousand brands and over 300 million consumers gather together forming an active online environment, which provides a high-quality and rich data basis for this study.

To ensure the stability and reliability of the research results, we have crawled data from a total of 461 livestream sessions from April 24, 2020 to May 29, 2020. Besides, the data were acquired from 166 different brands in various industries such as beauty, home appliances, snacks, and so on (e.g., NARS, Supor, Tao Li Food), involving 24 celebrities (e.g., Yilun Lin, Jie Ji, Ling Guan). Drawing on previous studies on the classification of product type and data coding methods (Opreana et al., 2015; Hazari et al., 2017), we randomly invited two marketing students who did not know the purpose of the experiment for data coding. For samples with inconsistent coding results, the two graduate students would discuss them with each other until a consistent coding result was obtained (Dubois et al., 2016; Hou et al., 2019).

### Variables and Model

Since the data came from multiple brands, which might interfere with the results, a mixed effect model was adopted in this study (Rust, 2005; Greven and Kneib, 2010). It shows as follows:

(1) $$L\,n\,\left(G\,r\,o\,w\,t\,h_{j}\right)=\beta_{0\,j}+\beta_{1}\,B\,C\,t\,y\,p\,e_{j}+\beta_{2}\,P\,r\,d\,c\,t\,t\,y\,p\,e_{j}+\beta_{3}\,B\,C\,t\,y\,p\,e_{j}\text{×}\,P\,r\,d\,c\,t\,t\,y\,p\,e_{j}+\beta_{4}\,L\,n\,\left(F\,a\,n\,s_{j}\right)+\beta_{5}\,L\,n\,\left(D\,u\,r\,a\,t\,i\,o\,n_{j}\right)+\beta_{6}\,L\,n\,\left(P\,r\,i\,c\,e_{j}\right)+\varepsilon_{j}$$

The fixed effect model:


(2) $$L\,n\,\left(G\,r\,o\,w\,t\,h_{j}\right)=\beta_{1}\,B\,C\,t\,y\,p\,e_{j}+\beta_{2}\,P\,r\,d\,c\,t\,t\,y\,p\,e_{j}+\beta_{3}\,B\,C\,t\,y\,p\,e_{j}\times P\,r\,d\,c\,t\,t\,y\,p\,e_{j}+\beta_{4}\,L\,n\,\left(F\,a\,n\,s_{j}\right)+\beta_{5}\,L\,n\,\left(D\,u\,r\,a\,t\,i\,o\,n_{j}\right)+\beta_{6}\,L\,n\,\left(P\,r\,i\,c\,e_{j}\right)+\varepsilon_{j}$$

The random effect model:


(3) $$\beta_{0\,j}=\gamma_{0}+\gamma_{1}\,B\,r\,a\,n\,d_{j}+\xi_{j}$$

#### Independent Variables

*BCtype*_*j*_ denotes the broadcaster type during the livestream session *j* (branded broadcaster = 0, celerity broadcaster = 1). *Prdcttype*_*j*_ represents the product type the broadcaster introduces during the livestream session *j* (utilitarian product = 0, hedonic product = 1). To avoid confounding effects, only the sessions that involve one product type (utilitarian/hedonic) were randomly selected in the study.

#### Dependent Variable

*Growth*_*j*_ stands for the cumulants of consumers who “newly follow” the brand during the livestream session *j*.

#### Control Variables

We selected three control variables from the community and product levels. *Fans*_*j*_ denotes the number of existing followers of a livestream brand community at the beginning of the livestream session *j*, which we considered as a control variable, because the number shows the popularity and value of a brand, and the more popular and valuable the brand account, the more new followers it may attract (Jin and Phua, 2014). Duration_j_ represents the duration of the livestream session *j*, which we considered as a control variable, owing to the cumulative effect over time, the longer the live shopping session, the more the number of followers. *Price*_*j*_ represents the price of the product in the livestream session *J.* Price is a key factor that the consumers consider during product and brand evaluation or purchase, which may further impact consumers’ following behavior (Beukeboom et al., 2015). Thus, we added it to our model as a control variable.

The descriptive statistics of the variables are shown in Table 2.

**TABLE 2:** Descriptive statistics of the variables.

Variable	Min.	Max.	Mean	*SD*
Broadcaster type	0.00	1.00	0.38	0.49
Product type	0.00	1.00	0.44	0.48
Cumulants of new followers	1.00	27700.00	3404.48	5164.47
Initial number of existing followers	6.00	16782500.00	1377283.46	2376264.45
Price	16.00	5099.00	350.30	685.11
Duration	99.00	87344.00	22005.57	14033.72

### Results

#### Broadcaster Type

Model 2 and Model 3 in Table 3 shows that broadcaster type has a significantly positive influence on the cumulants of consumers who “newly follow” the brand (Model 2: β = 0.617, *p*< 0.001; Model 3: β = 0.619, *p*< 0.001). In other words, celebrity broadcasters can attract more new followers than branded broadcasters, which supports **H1**.

**TABLE 3:** Regression results.

Independent variable		Dependent variable: Cumulants of new followers		
		Model 1	Model 2	Model 3
***Fixed effect***				
(Intercept)		0.016 (0.048)	0.007 (0.042)	−0.006 (0.042)
Broadcaster type			0.617*** (0.037)	0.619*** (0.037)
Product type			0.047 (0.039)	0.104* (0.043)
Broadcaster type × Product type				0.134** (0.044)
***Control variable***				
Initial number of existing followers		0.349*** (0.046)	0.279*** (0.040)	0.273*** (0.039)
Duration		0.123** (0.046)	0.062 (0.042)	0.063 (0.041)
Price		−0.071 (0.047)	−0.009 (0.040)	−0.004 (0.040)
***Random effect***				
Brand		Yes	Yes	Yes
	*Variance*	0.803	0.545	0.532
	*SD*	0.896	0.738	0.729
***AIC***		1270.04	1118.29	1115.40

①*p < 0.05, **p < 0.01, *** p < 0.001. ②Broadcaster type: 0 = branded broadcaster, 1 = celebrity broadcaster. ③ Product type: 0 = utilitarian, 1 = hedonic.

#### Product Type

As shown in Table 3, product type moderates the relationship between broadcaster type and the cumulants of consumers who “newly follow” the brand (Model 3: β = 0.134, *p*< 0.01). Specifically, hedonic (vs. utilitarian) products can enhance the influence of celebrity (vs. branded) broadcasters on the number of new followers, which supports the hypotheses **H2, H2a, H2b**.

### Robust Checks

To check the robustness of our results, we also conducted stratified models for utilitarian and hedonic product groups. Table 4 indicates that branded (vs. celebrity) broadcasters can promote (vs. restrain) the cumulants of consumers who “newly follow” the brand for utilitarian products (β = −0.551, *p*< 0.001), whereas celebrity broadcasters can facilitate the cumulants of new followers for hedonic products (β = 0.708, *p*< 0.001). These results are consistent with the hypotheses **H2a and H2b**.

**TABLE 4:** Results from moderation analyses.

Independent variables		Dependent variable: Cumulants of new followers	
		Utilitarian product	Hedonic product
***Fixed effect***			
(Intercept)		−0.067 (0.053)	0.092 (0.068)
Broadcaster type		−0.551*** (0.047)	0.708*** (0.059)
***Control variable***			
Initial number of existing followers		0.257*** (0.045)	0.303*** (0.085)
Duration		0.110* (0.053)	0.004 (0.065)
Price		−0.010 (0.046)	0.006 (0.081)
***Random effect***			
Brand		Yes	Yes
	*Variance*	0.536	0.527
	*SD*	0.732	0.726
***AIC***		686.666	443.136

①*p < 0.05, **p < 0.01, *** p < 0.001. ②Broadcaster type: 0 = branded broadcaster, 1 = celebrity broadcaster. ③ Product type: 0 = utilitarian, 1 = hedonic.

## Study 2: Laboratory Experiment for Main Effect and Moderating Effect Validation

In Study 1, we have tested **H1** and **H2** at the group level. But it should be noted that even though we have controlled variables such as initial number of existing followers, livestream duration, and product price, some confounding variables might still exist. Therefore, our subsequent Study 2 has controlled the confounding variables to enhance the internal validity and testify **H1** and **H2** at the individual level using intention to follow as the dependent variable.

### Participants and Design

The purpose of Study 2 is to test **H1** that consumers have a higher intention to follow the livestream brand community for celebrity broadcasters and **H2** that consumers have a higher intention to follow livestream brand community for celebrity (vs. branded) broadcasters selling hedonic (vs. utilitarian) products. This study used a 2 (broadcaster type: celebrity vs. branded) × 2 (product type: hedonic vs. utilitarian) between-subject design. A total of 212 undergraduate students (*N* = 120, 56.6% women; *M*_age_ = 22) participated in the experiment and received –Y10 as a reward after the complement of the experiment. Participants were randomly assigned to the four groups as mentioned above.

### Procedures

The subjects assigned in the celebrity group were required to write the name of a familiar celebrity but whom they did not dislike. Additionally, they were required to imagine that they were browsing a livestream shopping platform to buy a Bluetooth speaker. Then, they found the celebrity XX (the celebrity’s name the subject wrote down) was the broadcaster introducing a Bluetooth speaker of the brand TITO (an imaginary brand). Then, we would present a picture to show the description (utilitarian vs. hedonic attribute) of the Bluetooth speaker to manipulate the product type. Similarly, subjects assigned in the branded broadcaster group were required to imagine that a non-famous broadcaster hired by the brand was introducing the product (see Supplementary Material).

### Measurement

After the scenario simulation, the subjects were required to finish the questionnaire we provided.

First, we tested the subjects’ intention to follow the livestream brand community using two 7-point scale items adapted from Algesheimer et al. (2005); Belanche et al. (2014), and Casaló et al. (2017) (α = 0.826): “I would follow this brand livestream account (1 = ‘strongly disagree,’ 7 = ‘strongly agree’)” and “the probability I would follow this brand livestream account is (1 = ‘extremely low,’ 7 = ‘extremely high’).”

Second, to ensure the manipulation of broadcaster type working as intended, we tested the participants’ attitudes toward the broadcasters. The items for the celebrity broadcaster group were (1 = “strongly disagree,” 7 = “strongly agree”) as follows: “this broadcaster is famous,” “this broadcaster is attractive,” and “this broadcaster is appealing” (Friedman et al., 1978; Ohanian, 1991; Montoya et al., 2008) (α = 0.888). The items for the branded broadcaster group were as follows: “this broadcaster is from firm,” “this broadcaster is professional about the product,” and “I think this broadcaster can answer every question asked by the consumers” (Applbaum and Anatol, 1972) (α = 0.861). To verify the assumption on the non-significant difference between the two broadcaster types in perceived trustworthiness in section “The Influence of Broadcaster on Consumers’ Intention to Follow Livestream Brand Community*,”* we also tested it in the questionnaire. The items were “I think this broadcaster is honest” and “I think this broadcaster is reliable”(Sertoglu et al., 2014) (α = 0.883).

Third, the participants were required to rate the product they evaluated according to the hedonic or utilitarian attitudes (HED/UT) scale (Voss et al., 2003). Additionally, both hedonic (α = 0.996) and utilitarian attitudes (α = 0.995) were measured by three 7-point scale items.

### Results

#### Manipulation Check

The results showed that manipulation was successful. The participants were significantly more functional oriented in the utilitarian description group than the hedonic description group [*M*_utilitarian_group_ = 5.84, *SD* = 1.44, *M*_hedonic_group_ = 4.16, *SD* = 1.25; *F*_(1, 210)_ = 23.77, *p*< 0.01]. Similarly, the participants were significantly more hedonic oriented in the hedonic description group than the functional description group [*M*_hedonic_group_ = 5.54, *SD* = 1.31, *M*_utilitarian_group_ = 4.07, *SD* = 1.16; *F*_(1, 210)_ = 20.88, *p* < 0.01].

The manipulation of broadcaster type was also proven to be successful. The participants tended to have a higher evaluation of expertise of the broadcaster in the firm broadcaster group than the celebrity group [*M*_branded broadcaster_ = 5.21, *SD* = 1.22, *M*_celebritybroadcaster_ = 4.19, *SD* = 1.25; *F*_(1, 210)_ = 18.90, *p*< 0.01]. Likewise, the participants tended to have a higher evaluation of attractive of the broadcaster in the celebrity group than the branded broadcaster group [*M*_celebritybroadcaster_ = 5.45, *SD* = 1.34, *M*_branded broadcaster_ = 4.04, *SD* = 1.17; *F*_(1, 210)_ = 27.89, *p*< 0.01]. In addition, the participants reported no significant differences in trustworthiness between the celebrity and branded broadcaster groups [*M*_celebrity broadcaster_ = 4.79, *SD* = 1.44, *M*_branded broadcaster_ = 4.50, *SD* = 1.46; *F*_(1, 210)_ = 1.89, *p* > 0.05].

#### Intention to Follow the Livestream Brand Community

**FIGURE 2:**
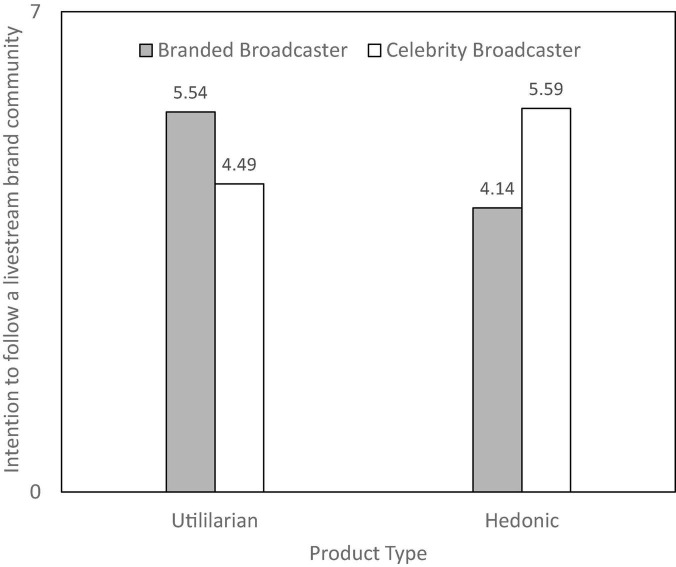
Study 2: Interaction of broadcaster type and product type on consumer’s intention to follow a livestream brand community.

An ANOVA test has revealed that celebrity broadcasters would promote a significantly higher intention to follow the livestream brand community of the consumers than branded broadcasters [*M*_celebritybroadcaster_ = 5.54 > *M*_branded broadcaster_ = 4.49; *F*_(1, 210)_ = 15.99, *p* < 0.001]. The results also revealed a significant interaction between broadcaster type and product type [*F*_(1, 208)_ = 11.46, *p* = 0.001], as illustrated in Figure 2. As predicted by H2, the participants have a significantly higher intention to follow the livestream brand community for the hedonic product facing celebrity broadcasters than branded broadcasters [*M*_celebrity broadcaster_ = 5.59 > *M*_branded broadcaster_ = 4.14; *F*_(1, 110)_ = 29.99, *p* < 0.001]. Participants have significantly higher intention to follow the livestream brand community for the utilitarian product when facing branded broadcasters than celebrity broadcasters [*M*_branded broadcaster_ = 5.47 > *M*_celebrity_ = 4.50; *F*_(1, 98)_ = 6.81, *p* < 0.05].

Study 2 initially demonstrates the main effect of broadcaster type and the moderating effect of product type on consumers’ following behavior. In support of **H1** and **H2**, the participants prefer celebrity broadcasters in general. In addition, they prefer branded broadcasters to celebrity broadcasters when making utilitarian product purchases, but their preference is to the contrary when making hedonic product purchases. However, the mechanism of the cause of the moderating effect remains unanswered. Therefore, in Study 3, we will delve into the proposed mediating effects of motivations.

## Study 3: Laboratory Experiment for Mediating Effect Test

In Study 3, we will test H3 that both informational and emotional value will mediate the effects of broadcaster type on the intention to follow livestream brand community, and the mediation is moderated by product type. Specifically, Study 3 aims to replicate the patterns in Study 2 and simultaneously shows how different motivations mediate the effects of broadcaster type on consumers’ intention to follow livestream brand community for different types of products.

### Study Design

A total of 126 undergraduate students (56% women; *M*_age_ = 20.7) were recruited for the experiment and a –Y10 reward was given after completing the experiment. The participants were randomly assigned to branded broadcaster or celebrity broadcaster scenarios where the broadcasters were selling a smartwatch using a utilitarian or hedonic description. The experiment procedure is similar to Study 2 (see Supplementary Material). First, the subjects were required to imagine the scenario in which a branded or celebrity broadcaster was recommending a smartwatch of the brand TIMIX (an imaginary brand) in a livestream shopping session. Then, they were provided with the experiment materials, including the picture of the product and the broadcaster’s descriptions of the product. After that, they were asked to finish a short questionnaire.

### Measurement

First, the participants were required to rate two items regarding their intention to follow the livestream brand community. Second, they rated two items about the informational value (Etemad-Sajadi and Ghachem, 2015): “the interaction with the broadcaster is useful” and “the interaction with the broadcaster is efficient” and two items about the emotional value (Etemad-Sajadi and Ghachem, 2015): “the interaction with the broadcaster is pleasant” and “the interaction with the broadcaster is interesting.” Last, they were asked to rate their attitudes toward product type and broadcaster type as in Study 2.

### Results

#### Manipulation Check

We successfully engendered differences in hedonic and utilitarian attitudes between the two groups. The participants have a higher rate in the functional categories in the utilitarian description group than the hedonic description group [*M*_utilitarian_group_ = 4.67, *SD* = 1.17, *M*_hedonic_group_ = 4.03, *SD* = 1.10; *F*_(1, 124)_ = 8.49, *p* < 0.01]. Similarly, the participants have a higher rate in the hedonic categories in the hedonic description group than the utilitarian description group [*M*_hedonic_group_ = 4.79, *SD* = 1.25, *M*_utilitarian_group_ = 4.25, *SD* = 1.13; *F*_(1, 124)_ = 4.28, *p* < 0.05].

The manipulation of the broadcaster type is also successful. The participants reported a higher rate in the professional categories in the firm broadcaster group than the celebrity group [*M*_branded broadcaster_ = 4.49, *SD* = 1.19, *M*_celebrity broadcater_ = 3.71, *SD* = 1.21; *F*_(1, 124)_ = 12.58, *p* = 0.001]. Additionally, the participants from the celebrity group tend to have a higher rate in attraction categories than the branded broadcaster group [*M*_celebrity broadcaster_ = 5.07, *SD* = 0.82, *M*_branded broadcaster_ = 3.31, *SD* = 1.11; *F*_(1, 124)_ = 101.81, *p*< 0.001]. In addition, the participants reported no significant differences in trustworthiness between the celebrity and branded broadcaster groups [*M*_celebrity broadcaster_ = 4.78, *SD* = 1.25, *M*_branded broadcaster_ = 4.73, *SD* = 1.45; *F*_(1, 124)_ = 0.17, *p* > 0.05].

#### Intention to Follow the Livestream Brand Community

The results are in line with those of Study 2 that the main effect of broadcaster type is significant [*M*_celebrity broadcaster_ = 5.18 > *M*_branded broadcaster_ = 4.06; *F*_(1, 120)_ = 19.84, *p* < 0.01]. A-two way ANOVA test has revealed a significant broadcaster type and product type interaction [*F*_(1, 125)_ = 8.29, *p* = 0.005]. The participants are more likely to follow the livestream brand community and become the fans of the brand when a hedonic product is recommended by a celebrity than a branded broadcaster [*M*_celebrity broadcaster_ = 5.52 > *M*_branded broadcaster_ = 3.69; *F*_(1, 59)_ = 27.44, *p* < 0.001]. The results also show that the participants are more likely to follow the livestream brand community and become the fans of the brand when a utilitarian product is recommended by a branded broadcaster than a celebrity broadcaster [*M*_branded broadcaster_ = 5.42 > *M*_celebritybroadcaster_ = 3.55; *F*_(1, 63)_ = 52.62, *p* < 0.001].

#### Moderated Mediation

We conducted a bootstrap for the moderated mediation analysis (Hayes, 2013). The results show that for utilitarian products, broadcaster type has a significantly negative effect on the informational value appeal (β = −1.57, SE = 0.31, *p* < 0.001), whereas the informational value in turn significantly affects the intention to follow livestream brand community (β = 0.38, SE = 0.11, *t* = 3.06, *p* = 0.004). Besides, the indirect effect analysis shows that the informational value mediates the influence of broadcaster type on the intention to follow livestream brand community (*Effect* = −0.56, *Boot SE* = 0.27, 95% CI: *LLCI* = −1.40, *ULCI* = −0.16), whereas the indirect effect of the hedonic value is not significant (*Effect* = 0.11, *Boot SE* = 0.16, 95% CI: *LLCI* = −0.50, *ULCI* = 0.16). These results indicate that a branded broadcaster introducing a utilitarian product will promote higher consumers’ intention to follow livestream brand community than a celebrity by meeting their informational value needs, supporting the hypothesis **H3a**.

The results also reveal that for hedonic products, broadcaster type has a significantly positive effect on the satisfaction of consumers’ informational value needs (β = 2.44, SE = 0.32, *t* = 7.15, *p* < 0.001), whereas the satisfaction of their emotional value needs will significantly affect their intention to follow livestream brand community (β = 0.39, SE = 0.11, *t* = 4.01, *p*< 0.001). Moreover, the indirect effect analysis show that the satisfaction of consumers’ emotional value needs mediates the influence of broadcaster type on consumers’ intention to follow livestream brand community (*Effect* = 0.93, *Boot SE* = 0.35, 95% CI: *LLCI* = 0.37, *ULCI* = 1.97), whereas the indirect effect of the satisfaction of the informational value is not significant (*Effect* = −0.08, *Boot SE* = 0.04, 95% CI: *LLCI* = −0.36, *ULCI* = 0.01). These results have indicated that a celebrity introducing a hedonic product will promote higher consumers’ intention to follow livestream brand community than a branded broadcaster by meeting their emotional value needs. Therefore, the hypothesis **H3b** is verified.

The results of Study 3 have implied that the interaction effect of broadcaster type and product type is mediated by consumer’s motivations. When consumers purchase hedonic (vs. utilitarian) products, a celebrity broadcaster (vs. branded broadcaster) will be more likely to meet their emotional (vs. informational) value and then lead to a higher intention to follow livestream brand community.

## Discussion

### Conclusion

This study has explored the relationship between two types of broadcasters and consumers’ intention to follow livestream brand communities. Moreover, the study has also investigated the effects of product type and informational and emotional value appeals on consumers’ intention to follow livestream brand communities. The investigations are mainly completed by analyzing the secondary data from the Taobao livestream platform and the data obtained from two laboratory experiments.

All hypotheses are tested positive. Overall, celebrity broadcasters have a more significant impact on consumers’ intention of following than branded broadcasters, indicating that social and experiential values are critical in livestream e-commerce (Xu et al., 2020; Fei et al., 2021; Li et al., 2021; Ming et al., 2021). Moreover, product type has a moderating effect on the effect of broadcaster type on consumers’ intention of following. Consumers are more likely to follow the livestream brand community when a celebrity (vs. branded) broadcaster recommends hedonic (vs. utilitarian) products. This is because celebrity (vs. branded) broadcasters can satisfy their emotional (vs. informational) value appeal. These results have shown that branded broadcasters are also of great significance in follower attraction, especially regarding utilitarian products. They have also revealed that the nature of consumer community participation is value acquisition.

### Theoretical Implications

The main theoretical contributions of this study are as follows.

First, it expands the research scope of the impact of livestream shopping on firm performance. At present, the discussions on livestream shopping mainly focus on the short-term economic benefits, such as the individual purchase intention at the consumer level (Hou et al., 2019; Ma and Mei, 2019; Zhang et al., 2019; Wongkitrungrueng and Assarut, 2020) and product sales at the enterprise level (Wongkitrungrueng et al., 2020). However, discussion on the long-term benefits of livestream shopping is limited. Building community and accumulating fans are the keys to the sustained profits and long-term development of livestream e-commerce and also the challenges for many firms. Many firms attempt to invite celebrity broadcasters to attract new followers but they are not effective every time. Therefore, some firms also try to cultivate branded broadcasters. To solve the long-term development problem and the dilemma of the broadcaster selection, this paper studied livestream shopping from the perspective of livestream brand community building and discussed the strategies for promoting the intention of following, which help to address the research gap.

Second, it may deepen our understanding of consumers’ following behavior in the livestream brand community. Based on previous studies on the social nature of livestream e-commerce and consumers’ motivations for community participation (Hou et al., 2019; Lim and Kumar, 2019; Zhang et al., 2019), we proposed a dual-path model to study consumers’ motivations for following and verified the mechanism how broadcaster type would influence consumers’ intention to follow the livestream brand community.

Third, it enriches the boundary of the impacts of different types of broadcasters. Different types of broadcasters should be combined with different product types to maximize their effects. For utilitarian (vs. hedonic) product, enterprises should choose branded (vs. celebrity) broadcasters to provide related value to attract fans.

### Practical Implications

This study also provides significant implications for the managers of livestream e-commerce markets.

This study may give implications to the sustainable profit issue in livestream e-commerce. Unlike the traditional TV market, which simply pursues short-term performance through promotions (e.g., discounting), livestream e-commerce brings enterprises more chances to achieve long-term performance. Enterprises should take advantage of the social nature of live e-commerce and seek to build solid relationships with customers for long-term success. One effective strategy is to build brands’ livestream communities and accumulate their own brand fans. Moreover, enterprises can rely on broadcasters to convey product information and brand values thus to attract potential consumers, thereby gaining more profits. However, livestream consumers also have emotional, experiential, and social needs. Therefore, corporations should also consider satisfying consumers’ demands by providing suitable broadcasters to maximize their business interests. The research has found that consumers may focus more on professional and reliable product information for brands selling utilitarian products. So, we recommend managers choose branded broadcasters to satisfy consumers’ informational needs. By contrast, as for hedonic products, managers could choose celebrity broadcasters to strengthen consumers’ joyful and exciting shopping experience to meet their emotional needs.

### Limitations

First, in this paper, we have discussed the broadcaster selection strategy for promoting the growth of the brand community size in livestream e-commerce. However, the paper has not touched upon the communication styles adopted by different types of broadcasters. For example, whether humor would have different impact across different types of broadcasters. Because humor may improve the consumers perceived pleasure but decrease their perceive professionalism. Second, our study only used the secondary data from transactional livestream shopping platform, and future studies can also try to examine the different impacts of broadcasters based on relationship-oriented livestream platforms (e.g., Weibo) or interest-oriented livestream platforms (e.g., Tiktok). Third, this research discussed only follower attraction; thus, future scholars can also investigate follower retention, which is another crucial problem in the long-term development of livestream brand communities.

## Data Availability Statement

The raw data supporting the conclusions of this article will be made available by the authors, without undue reservation.

## Ethics Statement

The studies involving human participants were reviewed and approved by the Economics and Management School, Wuhan University. The patients/participants provided their written informed consent to participate in this study.

## Author Contributions

WW conceived, designed the study, and wrote the manuscript. SZ and LL collected the data. SZ analyzed and interpreted the data. MH provided funding support. LW contributed to the revision process of the manuscript. All authors contributed to the article and approved the submitted version.

## Conflict of Interest

The authors declare that the research was conducted in the absence of any commercial or financial relationships that could be construed as a potential conflict of interest.

## Publisher’s Note

All claims expressed in this article are solely those of the authors and do not necessarily represent those of their affiliated organizations, or those of the publisher, the editors and the reviewers. Any product that may be evaluated in this article, or claim that may be made by its manufacturer, is not guaranteed or endorsed by the publisher.

## Footnotes

1
http://www.cnnic.cn/hlwfzyj/hlwxzbg/hlwtjbg/202109/t20210915_71543.htm
