# Supplementary material for: The Impact of Broadcasters on Consumer’s Intention to Follow Livestream Brand Community
Source: Front Psychol. 2022 Feb 3;12:810883. doi: 10.3389/fpsyg.2021.810883 (PMC8850974; doi:10.3389/fpsyg.2021.810883)
Supplement: Supplementary file 1 [file Data_Sheet_1.pdf]

Supplementary Material

1 Laboratory experiments materials

1.1 Study 2: Examples of group A1 & B1

We conducted a 2(Broadcaster type: branded vs. celebrity) ×2(Product type: utilitarian vs. hedonic) experiment and named the 4 groups as A1(celebrity broadcaster × hedonic product), A2(celebrity broadcaster × utilitarian product), B1(branded broadcaster × utilitarian product) and B2(branded broadcaster × hedonic product) respectively. The experiment materials (adapted from the real materials of Taobao Platform and Amazon) of group A1 and group B1 are as follows:

1.1.1 Group A1: Celebrity × Hedonic Product:

Hello, thank you very much for your time to participate this experiment. We are conducting a study on live e-commerce and want to invite you to answer some questions. You will get ￥10 as a reward after the valid answers are completed. There is no right or wrong answer, please just fill out the questionnaire according to your real feeling.

First, please write down the name of a celebrity you've been following on social media and you don’t hate her/him: _______________.

Then please imagine that, you are browsing a livestreaming shopping platform and planning to buy a Bluetooth speaker. And you find celebrity ____ (the name you wrote down above) is the broadcaster who is introducing a Bluetooth speaker from brand TITO. And he/she is describing the product as follows:

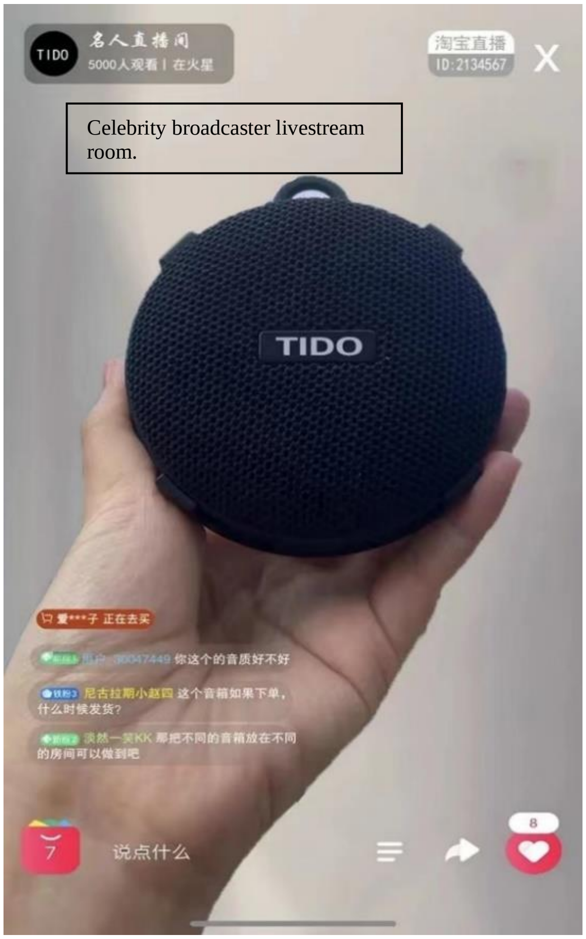

It’s so cute and has a classic design that has been refined and lightened, with effortlessly simple controls.

You can just build your own ecosystem by connecting multiple Sound Heroes enabled speakers together to amplify the listening experience.

You can get incredible sound at home, in the yard, or anywhere else imaginable. For example, you can enjoy high-quality music while on the move or taking a party with your friend anywhere.

I believe it can bring you more happiness for your life.

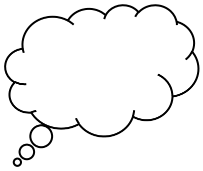

Figure 1: Scenario stimuli materials of group A1.

Then, please complete the questionnaire according to your real feeling.

1.1.2 Group B1: Branded broadcaster× Utilitarian Product

Hello, thank you very much for your time to participate this experiment. We are conducting a study on live e-commerce and want to invite you to answer some questions. You will get ￥10 as a reward after the valid answers are completed. There is no right or wrong answer, please just fill out the questionnaire according to your real feeling.

Then please imagine that, you are browsing a livestreaming shopping platform and planning to buy a Bluetooth speaker. And you find a salesperson from the brand TITO as the broadcaster who is introducing a Bluetooth speaker. And the salesperson is describing the product as follows:

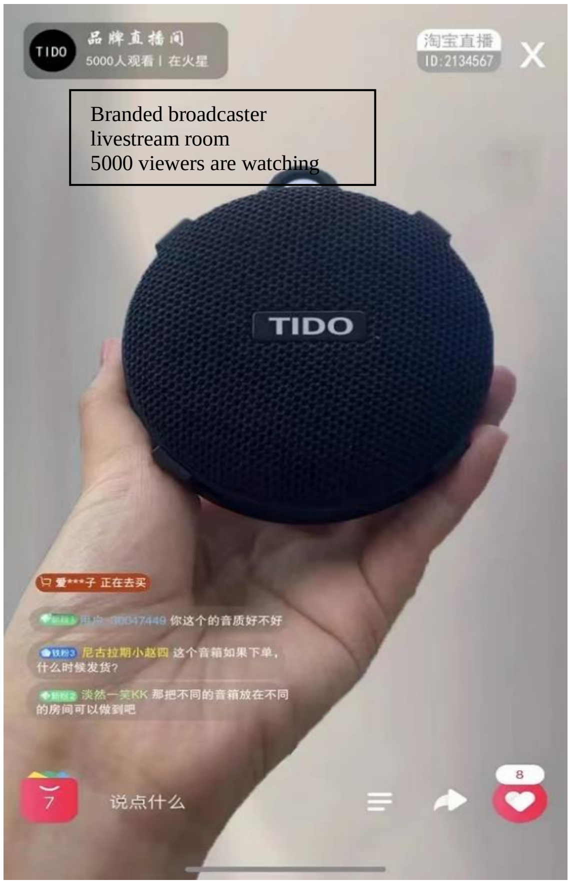

This Bluetooth speaker has a 12W of pure audio power with enhanced bass Thunders from dual neodymium drivers. An advanced digital signal processor ensures pounding bass and zero distortion at any volume.

It has an Anker’s world-renowned power management technology and a 5,200mAh Li-ion battery deliver a full day of sublime sound.

IPX7 protection safeguards against rain, dust, snow, and spills.

I believe it is a good choice with powerful function.

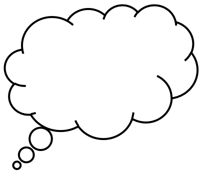

Figure 2: Scenario stimuli materials of group B1.

Then, please complete the questionnaire according to your real feeling.

1.2 Study 3: Examples of group C1 & D1

We conducted a 2(broadcaster type: branded vs. celebrity) ×2(product type: utilitarian vs. hedonic) experiment and named the 4 groups as C1(celebrity broadcaster × hedonic product), C2(celebrity broadcaster × utilitarian product), D1(branded broadcaster × utilitarian product) and D2(firm broadcaster × hedonic product) respectively. The experiment materials of group C1 and group D1 are as follows:

1.2.1 Group C1: Celebrity × Hedonic Product

Hello, thank you very much for your time to participate this experiment. We are conducting a study on live e-commerce and want to invite you to answer some questions. You will get ￥10 as a reward after the valid answers are completed. There is no right or wrong answer, please just fill out the questionnaire according to your real feeling.

First, please write down the name of a celebrity you've been following on social media and you don't hate her/him: _______________.

Then please imagine that, you are browsing a livestreaming shopping platform and planning to buy a smartwatch. And you find celebrity ____ (the name you wrote down above) is the broadcaster who is introducing a smartwatch from brand TIMIX. And he/she is describing the product as follows:

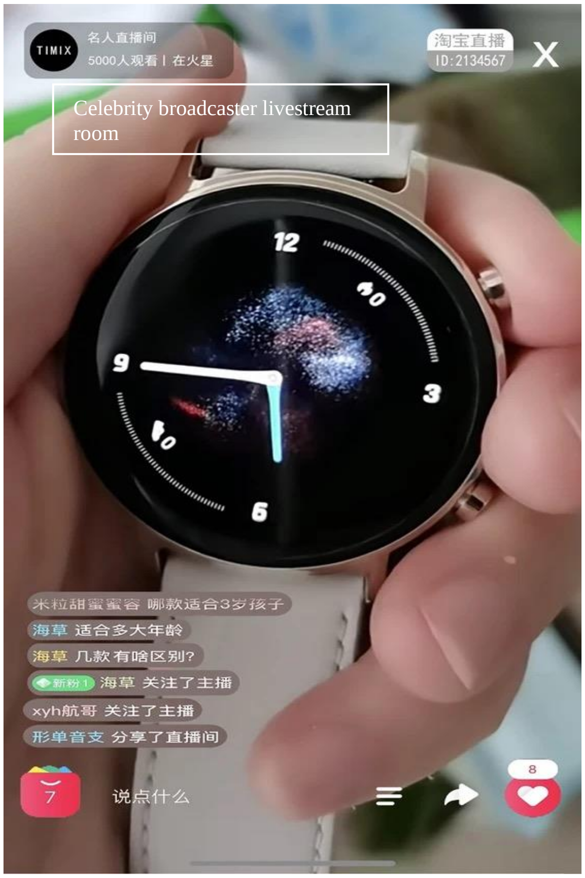

The smartwatch is no screen glare, screen brightness can be adjusted, so your eyes are not easily feel fatigued. The screen can be clearly seen even under sunlight. You can enjoy your music during your workouts and on the go.

You can also use the smartwatch to remote camera to record the cherished moment of your friends and your family. Just enjoy the time together with friends and family members.

It has personalized watch faces. You could set your favorite photo as it standby screen background. Different styles match your different moods.

The design is clean and simple. It can match your different outfits and make you look more fashionable and energetic.

Figure 3: Scenario stimuli materials of group C1.

Then, please complete the questionnaire according to your real feeling.

1.2.2 Group D1: Branded Broadcaster× Utilitarian Product

Hello, thank you very much for your time to participate this experiment. We are conducting a study on live e-commerce and want to invite you to answer some questions. You will get ￥10 as a reward after the valid answers are completed. There is no right or wrong answer, please just fill out the questionnaire according to your real feeling.

Then please imagine that, you are browsing a livestreaming shopping platform and planning to buy a smartphone. And you find a salesperson from the brand TIMIX who is as the broadcaster introducing a Bluetooth speaker. And the salesperson is describing the product as follows:

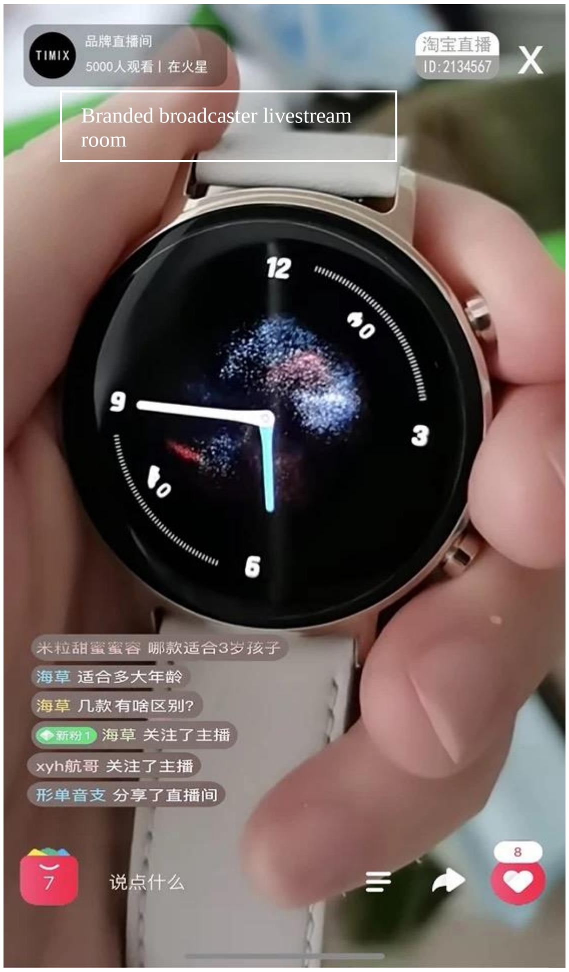

The 1.69" touch screen display with 300 PPI and 240*280 HD resolution, makes it clear and intuitive to understand your health and sports data.

This smartwatch includes more practical functions, monitor your real-time heart rate, blood oxygen, step count, calories burned, sleep stage, stress status data. Download and install free "TIMIX" APP, bind this smartwatch in your cell phone, you can receive alerts of SMS and Wechat, incoming calls on this watch. It will never let you miss any important things.

Large capacity and low power consumption design offers longer endurance. It comes with a magnetic charging USB cable, supports 7*24hr typical usage scenario after 2hr fully charged, standby time can reach more than 30 days.

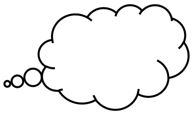

Figure 4: Scenario stimuli materials of group D1.

Then, please complete the questionnaire according to your real feeling.
